# Supplementary material for: The carcinine transporter CarT is required in Drosophila photoreceptor neurons to sustain histamine recycling
Source: eLife. 2015 Dec 14;4:e10972. doi: 10.7554/eLife.10972 (PMC4739767; doi:10.7554/eLife.10972)
Supplement: Figure 1—source data 1. — DOI: http://dx.doi.org/10.7554/eLife.10972.004 [file elife-10972-fig1-data1.docx]

| **Gene Name (abbreviation)** | **Gene Type (subtype)** | **Lines Tested** |
| --- | --- | --- |
| ABC transporter expressed in trachea (Atet) | ABC transporter (G) | VDRC100404 |
| ABCB7 | ABC transporter (B) | VDRC106039 |
| Alpha 3 glucosyltransferase (Alg10) | SLC36 | VDRC52402 |
| bedraggled (bdg) | SLC6 | VDRC44375 |
| bloated tubules (blot) | SLC6 | VDRC101083 |
| brown (bw) | ABC transporter (G) | VDRC101584 |
| Carcinine Transporter (carT) | SLC22 | VDRC101145 |
| CG1139 | SLC36 | VDRC102363 |
| CG1494 | ABC transporter (A) | VDRC30320 |
| CG1607 | SLC7 | VDRC105677 |
| CG1628 | SLC25 | VDRC109588 |
| CG1698 | SLC6 (Neutrient AA transporters) | VDRC101947 |
| CG1703 | ABC transporter (E) | VDRC105998 |
| CG1718 | ABC transporter (A) | VDRC105608 |
| CG1801 | ABC transporter (A) | VDRC100868 |
| CG1824 | ABC transporter (B) | VDRC106935 |
| CG2316 | ABC transporter (D) | VDRC41984; VDRC107342 |
| CG3156 | ABC transporter (B) | VDRC105646 |
| CG3164 | ABC transporter (G) | VDRC108413 |
| CG3790 | SLC22 | VDRC4667; VDRC108223 |
| CG4476 | SLC6 (Neutrient AA transporters) | VDRC109677 |
| CG4562 | ABC transporter (C) | VDRC106975 |
| CG4630 | SLC22 | BL61249; VDRC101254 |
| CG4794 | ABC transporter (A) | VDRC104215 |
| CG4822 | ABC transporter (G) | BL25894; VDRC105922 |
| CG4991 | SLC36 | VDRC108419 |
| CG5535 | SLC7 | VDRC107030 |
| CG5549 | SLC6 (Neurotransmitter transporter) | VDRC8222 |
| CG5592 | SLC22 | VDRC110489 |
| CG5789 | ABC transporter (C) | VDRC1204 |
| CG5853 | ABC transporter (G) | BL27668; VDRC100782 |
| CG6006 | SLC22 | BL52282; VDRC106513 |
| CG6052 | ABC transporter (A) | VDRC106738 |
| CG7255 | SLC7 | VDRC8373 |
| CG7346 | ABC transporter (G) | VDRC107236 |
| CG7491 | ABC transporter (A) | VDRC107135 |
| CG7627 | ABC transporter (C) | VDRC101084 |
| CG7708 | SLC1 | BL28613; VDRC101485 |
| CG7806 | ABC transporter (C) | VDRC2804 |
| CG7888 | SLC36 | VDRC37264 |
| CG8654 | SLC22 | BL57428; BL61850; VDRC100112 |
| CG8785 | SLC36 | VDRC4651 |
| **Gene Name (abbreviation)** | **Gene Type (subtype)** | **Lines Tested** |
| CG8850 | SLC6 (Neutrient AA transporters) | VDRC104098 |
| CG8908 | ABC transporter (A) | VDRC100472 |
| CG9270 | ABC transporter (C) | VDRC29961 |
| CG9281 | ABC transporter (E) | VDRC100263 |
| CG9330 | ABC transporter (E) | VDRC105156 |
| CG9413 | SLC7 | VDRC108867 |
| CG9663 | ABC transporter (G) | none available |
| CG9664 | ABC transporter (G) | VDRC42467 |
| CG9990 | ABC transporter (H) | VDRC107544 |
| CG10226 | ABC transporter (B) | VDRC108196 |
| CG10505 | ABC transporter (C) | VDRC107842 |
| CG10804 | SLC6 | BL29599; VDRC100400 |
| CG11069 | ABC transporter (G) | VDRC101080 |
| CG11147 | ABC transporter (H) | VDRC101601 |
| CG11897 | ABC transporter (C) | VDRC28260 |
| CG11898 | ABC transporter (C) | VDRC100660 |
| CG12531 | SLC7 | VDRC105771 |
| CG12943 | SLC36 | VDRC107119 |
| CG13248 | SLC7 | VDRC102635 |
| CG13384 | SLC36 | VDRC106698 |
| CG13646 | SLC1 | VDRC1571 |
| CG13743 | SLC1 | VDRC110773 |
| CG13793 | SLC6 | VDRC3669 |
| CG13794 | SLC6 | VDRC102583 |
| CG13795 | SLC6 | VDRC102250 |
| CG13796 | SLC6 | VDRC102516 |
| CG15279 | SLC6 (Neutrient AA transporters) | VDRC108759 |
| CG16700 | SLC36 | VDRC110058 |
| CG17119 | SLC1 | VDRC51127 |
| CG17646 | ABC transporter (G) | BL26008; VDRC100378 |
| CG30394 | SLC1 | VDRC3470 |
| CG31121 | ABC transporter (G) | VDRC100046 |
| CG31547 | SLC12 | VDRC105911 |
| CG31689 | ABC transporter (G) | VDRC102097 |
| CG31792 | ABC transporter (B) | VDRC107237 |
| CG31793 | ABC transporter (B) | VDRC8069 |
| CG31904 | SLC6 | VDRC34677 |
| CG32091 | ABC transporter (G) | VDRC107236 |
| CG33172 | ABC transporter (A) | VDRC100006 |
| CG33296 | SLC6 | VDRC50499 |
| CG33970 | ABC transporter (H) | VDRC101855 |
| CG34120 | ABC transporter (A) | VDRC101700 |
| **Gene Name (abbreviation)** | **Gene Type (subtype)** | **Lines Tested** |
| CG42269 | SLC22 | VDRC100344; VDRC101600 |
| CG42815 | ABC transporter (A) | none available |
| CG42816 | ABC transporter (A) | VDRC106262 |
| CG43066 | SLC6 | BL28069; VDRC101768 |
| CG43672 | ABC transporter (A) | VDRC48781 |
| CG43673 | ABC transporter (A) | none available |
| Dietary and metabolic glutamate transporter (dmGlut) | SLC6 (Neutrient AA transporters) | VDRC100034 |
| dopamine transporter (DAT) | SLC6 (Neurotransmitter transporter) | VDRC12082; VDRC106961 |
| Early gene at 23 (E23) | ABC transporter (G) | BL26252; VDRC105055 |
| Excitatory amino acid transporter (EAAT) 1 | SLC1 | VDRC109401 |
| Excitatory amino acid transporter (EAAT) 2 | SLC1 | BL40832; VDRC104371 |
| GABA transporter (dGAT) | SLC6 (Neurotransmitter transporter) | VDRC106638 |
| gap junction subunit innexin 1 (inx1) | innexin family | BL27283; VDRC7136 |
| gap junction subunit innexin 2 (inx2) | innexin family | BL29306; VDRC102194 |
| gap junction subunit innexin 3 (inx3) | innexin family | BL30501; VDRC108913 |
| genderblind (gb) | SLC7 | VDRC1261; VDRC1262 |
| Heavy metal tolerance factor 1 (Hmt-1) | ABC transporter (B) | VDRC37356 |
| inebrated (ine) | SLC6 | VDRC8880 |
| Juvenile hormone Inducing gene 21 (JhI-21) | SLC7 | VDRC45191; VDRC45193 |
| kazachoc (kcc) | SLC12 | BL34584; VDRC101742 |
| lethal (2) 03659 | ABC transporter (C) | VDRC100105 |
| Lithium-inducible SLC6 transporter (List) | SLC6 | VDRC109791 |
| Major Facilitator Superfamily Transporter 10 (MFS10) | Major Facilitator Superfamily | VDRC108145 |
| Major Facilitator Superfamily Transporter 15 (MFS15) | Major Facilitator Superfamily | VDRC106207 |
| Major Facilitator Superfamily Transporter 16 (MFS16) | Major Facilitator Superfamily | VDRC108635 |
| Major Facilitator Superfamily Transporter 17 (MFS17) | Major Facilitator Superfamily | VDRC109570 |
| Major Facilitator Superfamily Transporter 18 (MFS18) | Major Facilitator Superfamily | VDRC110554 |
| Major Facilitator Superfamily Transporter 3 (MFS3) | Major Facilitator Superfamily | VDRC107656 |
| minidiscs (mnd) | SLC7 | VDRC110217 |
| Monocarboxylate transporter 1 (Mct1) | Major Facilitator Superfamily | v106773 |
| Multi drug resistance 49 (Mdr49) | ABC transporter (B) | VDRC108327 |
| Multi drug resistance 50 (Mdr50) | ABC transporter (B) | VDRC51165 |
| Multi drug resistance 65 (Mdr65) | ABC transporter (B) | BL28664; BL35035 |
| Multidrug resistance protein 4 ortholog (Mrp4) | ABC transporter (C) | VDRC101221 |
| Multidrug-Resistance like Protein 1 (MRP) | ABC transporter (C) | BL38316; VDRC105419 |
| Neurotransmitter transporter-like (Ntl) | SLC6 (Neurotransmitter transporter) | VDRC102776 |
| Nutrient Amino Acid Transorter 1 (NAAT1) | SLC6 (Neutrient AA transporters) | VDRC106027 |
| organic cation transporter (Orct) | SLC22 | VDRC6782; VDRC47133; VDRC52658 |
| organic cation transporter 2 (Orct2) | SLC22 | VDRC48870; VDRC106681 |
| pathetic (path) | SLC36 | VDRC100519 |
| pixie (pix) | ABC transporter (E) | BL3160; VDRC109630 |
| Pmp70 | ABC transporter (D) | BL34349; VDRC110698 |
| **Gene Name (abbreviation)** | **Gene Type (subtype)** | **Lines Tested** |
| scarlet (st) | ABC transporter (G) | VDRC109793 |
| serotonin transporter (SerT) | SLC6 (Neurotransmitter transporter) | VDRC11346; VDRC100584 |
| Shaking B (shakB) | innexin family | BL27291; BL27292; VDRC24578 |
| slimfast (slif) | SLC7 | VDRC110425 |
| sodium chloride cotransporter 69 (Ncc69) | SLC12 | BL28682; VDRC106499 |
| Sulfonylurea receptor | ABC transporter (C) | VDRC104241 |
| Vesicular AcetylCholine Transporter (VAChT) | SLC18 | BL27684 |
| Vesicular GABA Transporter (VGAT) | SLC32 | BL41958 |
| Vesicular glutamate transporter (vGluT) | SLC17 | BL27538 |
| Vesicular monoamine transporter (Vmat) | SLC18 | BL31257 |
| white | ABC transporter (G) | VDRC30034 |
